# Blue-light treatment reduces spontaneous and evoked pain in a human experimental pain model

**DOI:** 10.1097/PR9.0000000000000968

**Published:** 2021-12-08

**Authors:** Anna Maria Reuss, Dominik Groos, Robert Scholl, Marco Schröter, Christian Maihöfner

**Affiliations:** a Department of Neurology, General Hospital Fürth, Faculty of Medicine, Friedrich-Alexander University Erlangen-Nuremberg (FAU), Germany; b Institute of Physiology and Pathophysiology, Faculty of Medicine, Friedrich-Alexander University (FAU), Erlangen-Nuremberg, Germany; c Department of Electrical Engineering and Mechanical Engineering, University of Applied Sciences, Bonn-Rhein-Sieg, Germany

**Keywords:** Blue light, Treatment, Therapy, Neuropathic pain, Hyperalgesia

## Abstract

**Introduction:**

Chronic pain is a frequent severe disease and often associated with anxiety, depression, insomnia, disability, and reduced quality of life. This maladaptive condition is further characterized by sensory loss, hyperalgesia, and allodynia. Blue light has been hypothesized to modulate sensory neurons and thereby influence nociception.

**Objectives:**

Here, we compared the effects of blue light vs red light and thermal control on pain sensation in a human experimental pain model.

**Methods:**

Pain, hyperalgesia, and allodynia were induced in 30 healthy volunteers through high-density transcutaneous electrical stimulation. Subsequently, blue light, red light, or thermal control treatment was applied in a cross-over design. The nonvisual effects of the respective light treatments were examined using a well-established quantitative sensory testing protocol. Somatosensory parameters as well as pain intensity and quality were scored.

**Results:**

Blue light substantially reduced spontaneous pain as assessed by numeric rating scale pain scoring. Similarly, pain quality was significantly altered as assessed by the German counterpart of the McGill Pain Questionnaire. Furthermore, blue light showed antihyperalgesic, antiallodynic, and antihypesthesic effects in contrast to red light or thermal control treatment.

**Conclusion:**

Blue-light phototherapy ameliorates pain intensity and quality in a human experimental pain model and reveals antihyperalgesic, antiallodynic, and antihypesthesic effects. Therefore, blue-light phototherapy may be a novel approach to treat pain in multiple conditions.

## Abstract 2

Supplemental Digital Content is Available in the Text.

Blue light reduces pain and shows antihyperalgesic effects in a human experimental pain model. Therefore, blue light may be a novel therapeutic approach for pain in multiple conditions.

## 1. Introduction

Maladaptive chronic pain is estimated to have a prevalence up to 10% and is often associated with anxiety, depression, insomnia, disability, and reduced quality of life.^48,51^ Chronic pain is one of the most common reasons why patients seek medical care.^20^ Generally, a multidisciplinary therapeutic approach is necessary. However, many treatment options are either only mildly analgesic or accompanied by deleterious side effects.^7,8,18,44,52^

Phototherapy is a nascent approach that has been shown to reduce chronic back, neck, and neuropathic pain without any significant side effects reported so far.^9,11,14,19,21,42^ In addition to low-level laser therapy, several preclinical and clinical studies have suggested the application of light-emitting diodes (LEDs) for the treatment of various conditions involving acute nociceptive and chronic pain.^10,12,16,27,30–32,40,45–47,53^ Although most studies have focused on the use of red and infrared light, few preclinical and clinical studies have explored the use of lower wavelength light.^22,33^ In a rat model of acute nociceptive pain, green and to a lesser extent blue light (BL) exhibited thermal analgesic effects.^33^ Interestingly, a large behavioral screen in zebrafish has revealed a naturally occurring light-sensitive ligand called optovin, which specifically activates transient receptor potential A1 (TRPA1) cation channels. On BL exposure, optovin transiently binds to TRPA1 through cysteine residues.^26^ This phenomenon has sparked the idea that BL could be used to modulate pain.^15^

Therefore, we hypothesized that prolonged exposure to BL may reduce spontaneous and evoked pain by modulating activated peripheral nociceptive fibers in human skin. To test this hypothesis, we used a well-described surrogate pain model in healthy volunteers using high-density transcutaneous electrical stimulation (HD-TES).^17,28,36^ Blue light, red light (RL), or thermal control (TC) treatment was applied through custom-made (home-built) collars in a cross-over fashion. The reduction of numeric rating scale (NRS) pain scores after 1-hour light treatment was assessed as the primary outcome.

## 2. Materials and Methods

### 2.1. Subjects

A total of 30 healthy volunteers were recruited. All participants were required to be at least 18 years old. The exclusion criteria were dermatological diseases and regular use of analgesics. The mean habitual life quality was assessed by 7 questions according to the Marburg questionnaire of general life quality.^6^ The depression and anxiety stress scale was assessed by 21 questions according to Nilges and Essau.^37^ Detailed information about the subjects is presented in Table 1.

**Table 1:** Characteristics of study participants.

Factor	Mean	SEM	%
Age	25.53	1.43	—
BMI	22.60	0.45	—
Habitual life quality	4.78	0.10	—
DASS	0.43	0.05	—
Menstrual cycle	12.46	2.55	—
Partnership: single	—	—	53.33: 46.67
Contentment with relationship status	—	—	90

Assessment of age, body mass index (BMI), habitual quality of life, depression, anxiety stress scale (DASS), menstrual cycle, relationship status, and contentment with relationship status. Means are given ±SEM (n = 30).

The aim of the study and different treatments were explained to the subjects only in a broad sense. Participants were blinded for the light treatments applied. All volunteers signed an informed consent form before the start of the experiment. The study was conducted according to the Declaration of Helsinki on biomedical research involving human subjects (Edinburgh amendment). The proposal of the study was approved by the Local Ethics Committee of the University of Erlangen–Nuremberg.

Subjects were instructed not to take analgesics when the experiments took place. The study was conducted in a cross-over design by dividing the subjects into 3 groups, each consisting of 10 test persons (50% male and 50% female). Randomization was achieved by stratified random allocation according to sex using Excel (Microsoft, Redmond), and each group started with a different treatment condition. Afterwards, the 2 remaining treatment conditions were applied, each with an interval of 4 weeks.

The study was performed in the Department of Neurology, General Hospital Fürth, Germany, in summer 2016.

### 2.2. Experimental design

**Figure 1.**
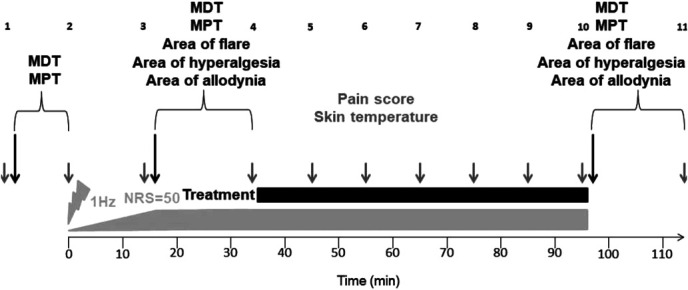
Experimental protocol. Pain was induced on the volar forearm through transcutaneous electrical stimulation (gray bar) using an impulse generator with a frequency of 1 Hz. The current was gradually increased during the first 15 minutes until NRS = 50, and kept constantly until the end of stimulation. Pain status (gray arrows) was assessed qualitatively and quantitatively before and after QST as well as every 10 minutes during treatment. At the same time, skin temperature (gray arrows) of the test and control volar forearm was recorded using a thermal camera. For QST (black arrows), MDT and MPT were assessed before electrical stimulation as well as before and after light treatment (black bar) together with areas of flare, hyperalgesia, and allodynia. MDT, mechanical detection threshold; MPT, mechanical pain threshold; NRS, numeric rating scale; QST, quantitative sensory testing.

The experimental design of the study is illustrated in Figure 1. At the beginning of every experiment, pain scoring was assessed qualitatively and quantitatively on a 100-point NRS, ranging from 0 = no pain to 100 = maximum of tolerable pain. Pain scoring was performed before and after quantitative sensory testing (QST) and every 10 minutes during treatment. At the same time points, skin temperature of the test and control volar forearm was recorded using a thermal camera to assess the temperature profile during the experiment. Pain was induced on the test arm using HD-TES until NRS = 50 was reached. Treatment with BL, RL, or TC was performed for 1 hour. Before electrical stimulation, QST was performed by assessing the mechanical detection threshold (MDT) and mechanical pain threshold (MPT). These parameters were further determined before and after treatment together with measurement of the area of flare, mechanical hyperalgesia, and allodynia.

### 2.3. Electrical induction of pain

For HD-TES, 2 self-adhesive electrodes (2 mm × 5 mm) were placed on the skin of the dominant hand volar forearm with a distance of 4 mm between them. Monophasic, rectangular electrical pulses with a frequency of 1 Hz and a voltage of 400 mV were applied using a pulse generator (Digitimer S7; Hertfordshire, UK). The current was gradually increased during the first 15 minutes until NRS = 50 with a predefined maximum of 40 mA and then kept constant until the end of stimulation. To ensure identical baseline conditions before the application of distinct treatments, current profiles were assessed during the adjustment period of electrical stimulation.

### 2.4. Quantification and qualification of induced pain

For quantification of induced pain, subjects were asked to rate their pain using the NRS, ranging from 0 = no pain to 100 = maximum tolerable pain. To measure pain perception in either direction, HD-TES intensity was adjusted to NRS = 50. To account for individually varying pain adaptation, HD-TES intensity was readjusted after 15 minutes of stimulation to NRS = 50 if it had deviated from NRS = 50. Subsequently, subjects were asked in regular intervals of 10 minutes to rate pain perception on a NRS 0 to 100.

For qualification of induced pain, subjects were asked to describe the quality of pain according to the German counterpart of the McGill Pain Questionnaire.^13,34^

### 2.5. Quantitative sensory testing

To determine MDT,^41^ a standardized set of von Frey hairs (Optihair set; Marstock Nervtest, Heidelberg, Germany) was used, exerting force between 0.25 mN and 256 mN graded by a factor of 2 on a flat contact area of <1 mm^2^ with a contact time of 1 second on the skin around the electrodes. Test persons were asked whether they felt the force or not.

To determine MPT,^41^ a standardized set of pinprick punctate probes with 7 custom-made (in-house built)-weighted stimulus intensities between 8 mN and 512 mN graded by a factor of 2 was used. Pinprick punctuate probes were placed on a flat contact area of 0.2 mm diameter with a contact time of 1 second on the skin around the electrodes. Test persons were asked whether they perceived the stimulus as sharp or not. Applying a “modified method of limits,” the geometric mean of MDT and MPT was calculated from 5 lines of ascending and descending stimulus intensities.

To determine the areas of flare, mechanical hyperalgesia, and allodynia,^36^ 8 linear rows separated by angles of 45°, running from the center around the electrodes to the periphery and consisting of points with each 1-cm distance were drawn using a template.

The border of the red flare was determined by counting the points' distances from the center for each row.

Hyperalgesia was measured by using a 256-mN pinprick punctate probe on a flat contact area of 0.2 mm diameter with a contact time of 1 second on the skin, going from point to point from the periphery to the center for each row.

Allodynia was measured using a cotton wool that stroked the skin for a length of 1 cm and with a force of 3 mN, extending from the periphery to the center of each row.

Areas of flare, hyperalgesia, and allodynia were calculated by summing up the calculated areas of all triangles in the template, which delineated the borders of the measured flare, hyperalgesia, and allodynia, respectively.

### 2.6. Light treatment

Treatment was performed with custom-made (in-house built) opaque collars, emitting either 450 nm BL or RL of 630 nm wavelength. The irradiation unit of the collar consisted of 150 surface mount device LEDs, reaching an optical output power of 0.8 Watt. As TC, an opaque collar was designed producing heat only. The opaque collars were placed around the electrically stimulated forearm before switching them on so that the test person could not see the illumination. For temperature measurements, the light was switched off to ensure subjects' blindness. Light treatment was applied with an initial intensity of 50%. When skin temperature rose above 40°C, the intensity was decreased to 25% to avoid blisters and then reincreased when skin temperature fell again below the 40°C threshold.

### 2.7. Measurement of skin temperature

Skin temperature was measured on the test and control volar forearm using a thermal camera (FLIR T640; Frankfurt on the Main, Germany).

### 2.8. Data analysis

Data analysis was performed using Excel (Microsoft), GraphPad Prism 5 (La Jolla), and MATLAB (MathWorks, Natick). Groups were pooled and analyzed in a blinded way. Data are represented as mean with SD or median with interquartile range (25th and 75th percentiles) with extreme data points (boxplot whiskers) and outliers (“+” symbols). Since data did not correspond to a Gaussian distribution, comparisons were evaluated statistically either using nonparametric 1-way analysis of variance for repeated measures (Friedman test) with the Dunn multiple comparison posttest or aligned rank transform analysis of variance for nonparametric factorial analysis with repeated measures. Significance was assumed for *P* < 0.05.

## 3. Results

### 3.1. Current and temperature profiles

**Figure 2.**
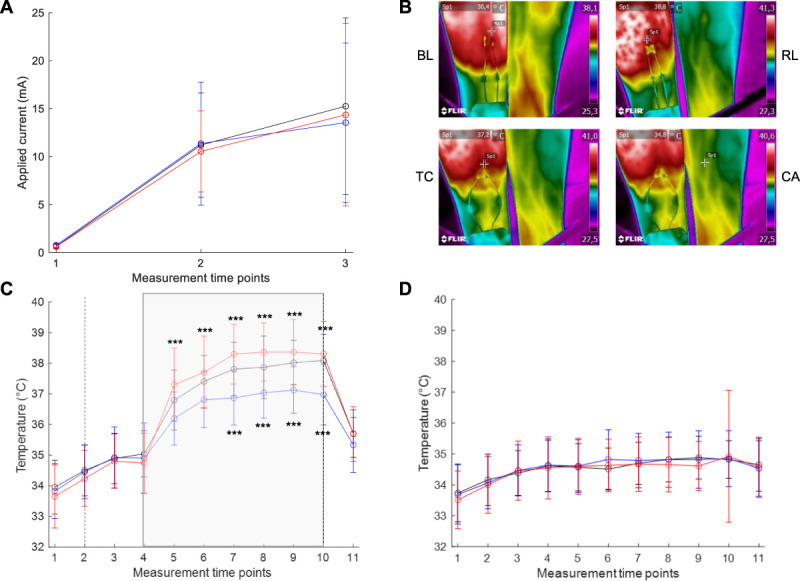
Applied current and temperature profiles. (A) Electrical currents in mA applied to subjects before treatment either with thermal control (TC; dark line), blue light (BL; blue line), or red light (RL; red line) at t = 1 the detection threshold, t = 2 at NRS = 50, and t = 3 after 15 minutes, before currents were kept constantly and treatment was started. Means are given ±SD (n = 30). (B) Representative photographs of the test person's volar right forearm under treatment with TC, BL, and RL as well as of the unstimulated and untreated left control arm (CA) detected by a thermal camera. (C) The mean temperature profiles in °C of subjects' test arm treated with TC, BL, or RL. Intervals between time points from 1 to 10 comprise 10 minutes, and the interval between the time points 10 and 11 comprises 20 minutes. *t* = 5 represents the time point 10 minutes after treatment, and *t* = 11 the time point after completion of electrical stimulation and treatment. For statistical analysis, data were aligned rank transformed, and 2-way ANOVA with the Bonferroni posttest was calculated. ***P < 0.001. Means are given ±SD (n = 30). (D) The mean temperature profiles in °C of subjects' control arm. Analysis was the same as for (C). ANOVA, analysis of variance.

For all 3 treatment groups (TC, BL, and RL), the mean current profiles were similar before treatment application (Fig. 2A), indicating the same baseline conditions.

Temperature profiles revealed that electrical stimulation alone did not generate a warming effect in contrast to all treatments, which increased temperature by about 2 to 4°C compared to the unstimulated and untreated control arm (Fig. 2B–D). Interestingly, this effect was stronger for RL than for TC and weakest for BL throughout the treatment period with no differences among the 3 groups after treatment termination. The order of treatments across subjects did not have any impact on temperature profiles (Supplemental Table 1, available at http://links.lww.com/PR9/A136).

### 3.2. Impact of light treatment on pain sensation

**Figure 3.**
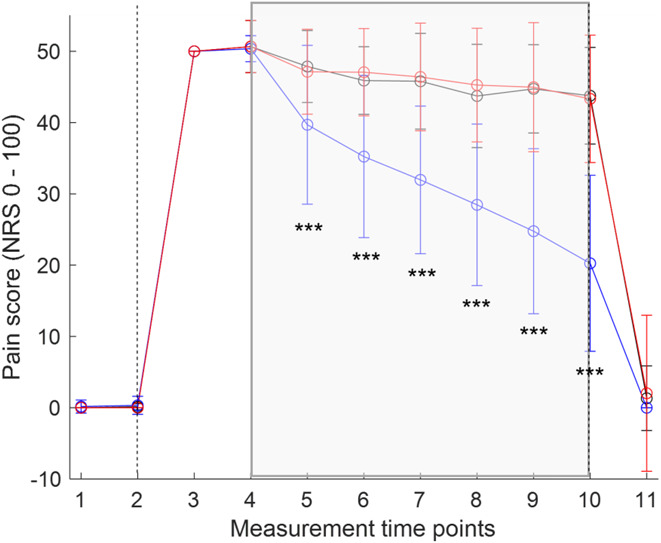
Impact of treatment on pain sensation in the test arm. NRS pain scoring under treatment with TC (black line), BL (blue line), and RL (red line). Intervals between time points from 1 to 10 comprise 10 minutes, and the interval between the time points 10 and 11 comprises 20 minutes. Start (*t* = 2) and termination (*t* = 10) of electrical stimulation are indicated by vertical dashed lines. Treatment periods are indicated by a colored gray box. For statistical analysis, data were aligned rank transformed, and 2-way ANOVA with the Bonferroni posttest was calculated. ***P < 0.001. Means are given ±SD (n = 30). ANOVA, analysis of variance; NRS, numeric rating scale.

From all treatments, only BL reduced NRS pain scores significantly (Fig. 3). This effect was visible as early as 10 minutes after initiation of exposure and continued to increase over the course of treatment, leading to a reduction in NRS pain scores by about half after 60 minutes. Twenty minutes after termination of electrical stimulation and treatment, pain scores returned close to 0 independent of treatment. However, there were 10% nonresponders with no significant decrease in NRS pain scores after BL treatment included in the analysis. NRS pain scores remained negligible in the unstimulated, untreated control arm during the entire course of experiment (Supplemental Figure 1, available at http://links.lww.com/PR9/A136). Moreover, the order of light treatment did not have any significant impact on NRS pain scores reported for the test arm (Supplemental Table 2, available at http://links.lww.com/PR9/A136).

Qualitative pain sensation showed marked individual variation. However, during distinct treatments, qualitative pain sensation differed depending on the condition applied (Table 2; Supplemental Figure 2, available at http://links.lww.com/PR9/A136). Of note, during BL treatment, participants sensed pain as less hot, tingling, stabbing, and throbbing than during RL and TC treatment.

**Table 2:** Statistical comparison of test arm pain quality dependent on treatment.

Pain quality	TC vs BL Difference	TC vs RL Difference	BL vs RL Difference
Itchy	0.8333*	1.167*	0.333*
Pressing	−0.6667*	0.000*	0.6667*
Dull	−2.500*	3.667†	6.167§
Burning	−4.167‡	−1.167*	3.000*
Tingling	−4.833§	−0.8333*	4.000‡
Beating	1.000*	1.500*	0.500*
Hot	−11.83§	8.500§	20.33§
Pulling	−8.667§	−8.333§	0.333*
Stabbing	−11.00§	−3.500†	7.500§
Throbbing	−9.833§	−6.000§	3.833†

Statistical analysis of pain quality under treatment with TC, BL, and RL. Two-way ANOVA was calculated with the Bonferroni posttest.

* *P* > 0.05.

† *P* < 0.05.

‡ *P* < 0.01.

§ *P* < 0.001.

BL, blue light; RL, red light; TC, thermal control.

### 3.3. Impact of light treatment on quantitative sensory testing parameters

Independent of treatment group, HD-TES increased MDT and MPT to the same extent in the test arm (Supplemental Figure 3A and B, available at http://links.lww.com/PR9/A136), but not in the control arm (Supplemental Figure 3C and D, available at http://links.lww.com/PR9/A136). This result indicates that HD-TES led to hypesthesia and reduced sensitivity for sharp stimulus discrimination.

**Figure 4.**
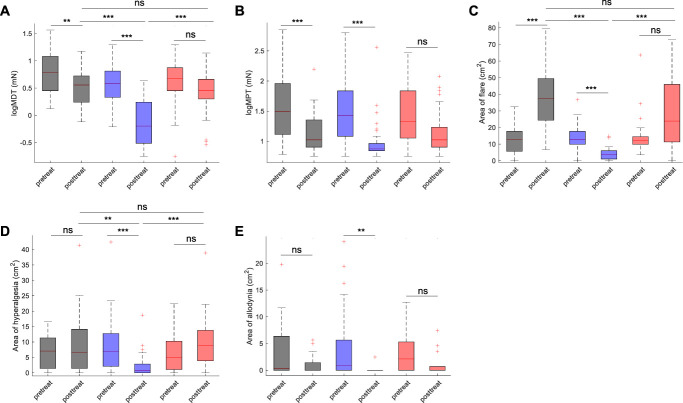
Impact of treatment on QST parameters in the test arm. (A) Logarithmic MDT in mN (n = 30) before (pretreat) and after (posttreat) treatment with TC (black boxes), BL (blue boxes), and RL (red boxes). All data are represented as median (red line) with interquartile range (box edges represent 25th and 75th percentiles) with extreme data points (boxplot whiskers) and outliers (“+” symbols). Nonparametric 1-way ANOVA for repeated measures (Friedman test) was calculated with the Dunn posttest. ^ns^*P* > 0.05, **P < 0.01, and ***P < 0.001. (B) Logarithmic MPT in mN (n = 30) before (pretreat) and after (posttreat) treatment with TC, BL, and RL, normalized to the time point before electrical stimulation. One-way ANOVA (Friedman test) with the Dunn posttest. ^ns^*P* > 0.05, ***P < 0.001. (C) Area of flare in cm^2^ (n = 30) before (pretreat) and after (posttreat) treatment with TC, BL, and RL. Nonparametric 1-way ANOVA for repeated measures (Friedman test) was calculated with the Dunn posttest. ^ns^*P* > 0.05, ***P < 0.001. (D) Area of hyperalgesia in cm^2^ (n = 30) before (pretreat) and after (posttreat) treatment with TC, BL, and RL. Nonparametric 1-way ANOVA for repeated measures (Friedman test) was calculated with the Dunn posttest. ^ns^*P* > 0.05, **P < 0.01, ***P < 0.001. (E) Area of allodynia in cm^2^ (n = 30) before (pretreat) and after (posttreat) treatment with TC, BL, and RL. Nonparametric 1-way ANOVA for repeated measures (Friedman test) was calculated with the Dunn posttest. ^ns^*P* > 0.05, **P < 0.01. ANOVA, analysis of variance; MDT, mechanical detection threshold; MPT, mechanical pain threshold; QST, quantitative sensory testing.

Induced hypesthesia decreased under BL and TC treatment, whereas RL did not lead to a significant reduction (Fig. 4A). However, hypesthesia reduction was significantly stronger on BL exposure.

Similar to MDT, MPT decreased under BL treatment and less stronger under TC treatment, whereas RL treatment failed to reduce MPT (Fig. 4B).

For all 3 groups, a red flare was induced by HD-TES. Interestingly, although the area of flare further increased under TC and RL treatment, it decreased under BL treatment (Fig. 4C).

Similarly, the area of mechanical hyperalgesia induced by HD-TES only diminished under blue light treatment (Fig. 4D). This effect was highly significant, although hyperalgesia could not be induced in all subjects.

Similarly, allodynia was observed only in 11 individuals. However, allodynia was significantly reduced only under BL treatment (Fig. 4E).

On the control arm, neither HD-TES nor light treatment affected QST parameters (Supplemental Figure 4, available at http://links.lww.com/PR9/A136). Except for the area of flare between BL and RL treatment, there was no significant difference observed for QST parameters based on the order of applied treatments among subjects, indicating marginal stratification of the data (Supplemental Table 3, available at http://links.lww.com/PR9/A136).

## 4. Discussion

In this study, we found that BL treatment substantially reduced various parameters of spontaneous and evoked pain in a human experimental pain model in contrast to RL and TC.

### 4.1. Potential mechanisms of blue-light treatment

Short-wavelength BL has been shown to specifically activate TRPA1 cation channels in zebrafish through transient binding of the light-sensitive ligand optovin.^26^ Low-intensity light of the same wavelength induces painful sensation, when applied to the skin of healthy volunteers.^5^ Furthermore, BL up to 460 nm wavelength is sufficient to activate human TRPA1 and already sensitized TRPV1 channels in transfected HEK 293 T cells.^4,5^ On activation, TRPA1 and TRPV1 channels located at unmyelinated C and small myelinated Aδ fibers induce the release of proinflammatory cytokines.^35,38^ The neuropeptides substance P and calcitonin gene–related peptide lead to protein extravasation and vasodilatation, which are the key mechanisms in neurogenic inflammation and nociceptor sensitization.^5,23^ Accordingly, a crucial role of TRPA1 and TRPV1 in neurogenic inflammation and chronic pain has been suggested.^3,5,24,50^ Interestingly, reactive oxygen species, which also activate TRPA1, lead to photosensitization and augment pain response to BL treatment.^5^

Brief stimulation with BL has been demonstrated to be nociceptive; however, we have observed an antinociceptive effect for extended periods of illumination in a human surrogate model of chronic pain. On the one hand, this finding might be explained by the excitation status of nociceptive fibers. Owing to electrical stimulation, nociceptive fibers are constantly depolarized, resembling conditions of chronic pain. Additional activation of already sensitized TRPA1 and TRPV1 cation channels by BL illumination may result in the efflux instead of influx of cations, leading to hyperpolarization of excited nociceptive fibers. Alternatively, extended BL illumination may lead to a desensitization of nociceptive fibers as shown for various TRP agonists.^1,2,25,43^ Finally, BL has been shown to activate antioxidative capacities of human skin fibroblasts, which may mediate further antinociceptive effects.^29,39^ However, the optimal extent and time scale required for BL to affect nociception, especially in patients with chronic pain, requires further investigation.

### 4.2. Rescue of hypesthesia and stimulus discrimination under blue-light treatment

In addition to pain induction, HD-TES caused hypesthesia and decreased the potential to discriminate sharp stimuli, thus mimicking sensory loss observed in patients with chronic pain.^49^ Blue-light treatment significantly improved hypesthesia and sharp stimulus discrimination in contrast to RL treatment. Interestingly, TC resulted in modest improvement, indicating that the warming effect alone may already be beneficial.

### 4.3. Potential limitations of the study

Although we obtained promising results with BL treatment in our human experimental pain model, some potential limitations need to be considered. First, although the participants were blinded to the treatment applied during each session, they were not explicitly asked to guess to avoid generating expectations in participants. Therefore, we cannot evaluate whether participants attempted to surmise which treatment was being applied during the individual sessions. Unlike participants, the examinator was not blinded for the light treatment.

Moreover, although we expected skin temperature to rise on the test arm under light treatment, there were significant differences between RL and BL. This phenomenon may be due to variable penetration depths and thus different quantities of excited molecules within the tissue. Of note, BL with the lowest penetration depth increased temperature much less than RL with the greatest penetration depth.

Although the area of flare decreased under BL exposure, it intensified under RL and TC treatment. On the one hand, stronger heating by RL and TC may have led to the increased flare. On the other hand, increased flare may be due to neurogenic inflammation induced by HD-TES and not suppressed by TC or RL treatment. Interestingly, although skin temperature rose significantly under BL exposure, the area of flare decreased. This finding suggests that BL attenuates neurogenic inflammation independent of skin temperature.

Finally, although LED-based BL phototherapy effectively reduced spontaneous and evoked pain, 10% of test subjects did not respond to BL treatment. This phenomenon needs to be considered for potential further testing in patients with chronic pain, who represent a much more heterogenous population.

### 4.4. Clinical relevance of the study

The use of LED-based phototherapy for treating acute nociceptive and chronic pain disorders has increased significantly over the past years because of its low costs, ease of application, and lack of side effects. In particular, red and infrared LEDs have proven to be valuable tools in managing acute and chronic pain in conditions such as knee osteoarthritis,^12^ back pain,^30,31^ temporomandibular joint disorder,^27,45^ orthodontic tooth separation,^16^ and childbirth.^46,47^ Moreover, green-light treatment has revealed promising results in treating chronic and episodic migraine.^33^ However, to date, BL has not been applied for pain treatment in clinical settings.

In our preclinical study, we show that extended exposure to BL leads to significant analgetic, antihyperalgesic, and antiallodynic effects without any evident side effects. These results pave the way to test LED-based BL phototherapy in clinical settings. This novel treatment approach may extend the spectrum of available therapies for acute and chronic pain disorders. Future studies should attempt to elucidate any potential differences in the effects of BL treatment on acute vs chronic pain states as well as the origin of nociceptive, nociplastic, and neuropathic pain.

## 5. Conclusion

In this study, BL treatment significantly reduced pain intensity and quality in a human experimental pain model. Furthermore, BL showed significant antihyperalgesic, antiallodynic, and antihypesthesic effects. Therefore, BL phototherapy may be a novel approach to treat pain in multiple conditions.

## Disclosures

A. M. Reuss, D. Groos, M. Schröter, and R. Scholl declare no competing financial or nonfinancial conflict of interest. In the past 3 years, C. Maihöfner has worked as a consultant and speaker for the following companies: Allergan, Bionorica, Biotest, Grünenthal, GSK, Lilly, Novartis, and Daiichi Sankyo. The present work was performed in fulfillment of the requirements for obtaining the degree “Dr. med.” by Anna Maria Reuss.

## Appendix A. Supplemental digital content

Supplemental digital content associated with this article can be found online at http://links.lww.com/PR9/A136.

## Supplementary Material

SUPPLEMENTARY MATERIAL
