# Supplementary material for: Blue-light treatment reduces spontaneous and evoked pain in a human experimental pain model
Source: Pain Rep. 2021 Dec 8;6(4):e968. doi: 10.1097/PR9.0000000000000968 (PMC8660004; doi:10.1097/PR9.0000000000000968)
Supplement: SUPPLEMENTARY MATERIAL [file painreports-6-e968-s001.pdf]

Blue light treatment reduces spontaneous and evoked pain in an experimental neuropathic pain model

Supplementary Material

Anna Maria Reuss, Dominik Groos, Robert Scholl, Marco Schröter, Christian Maihöfner

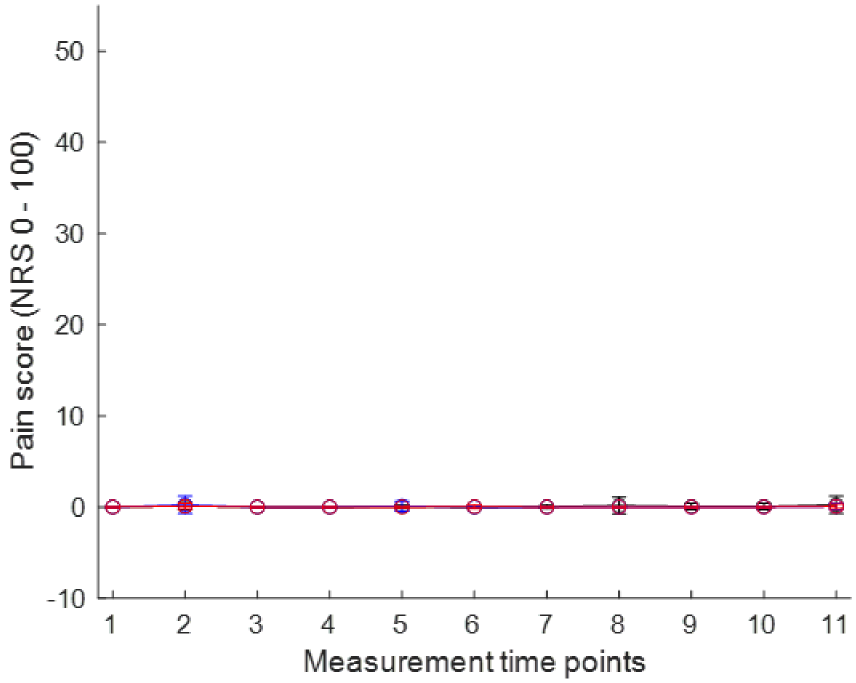

Supplementary Figure 1. Impact of treatment on pain sensation in control arm.

NRS pain scoring of unstimulated and untreated control arm while test arm was treated with TC (black line), BL (blue line), and RL (red line). Intervals between time points from 1 to 10 comprise 10 min, the interval between the time points 10 and 11 comprises 20 min. For statistical analysis data were aligned-rank transformed and two-way-ANOVA with Bonferroni posttest was calculated. $^{ns}p > 0.05$. Means are given ± SD ($n = 30$).

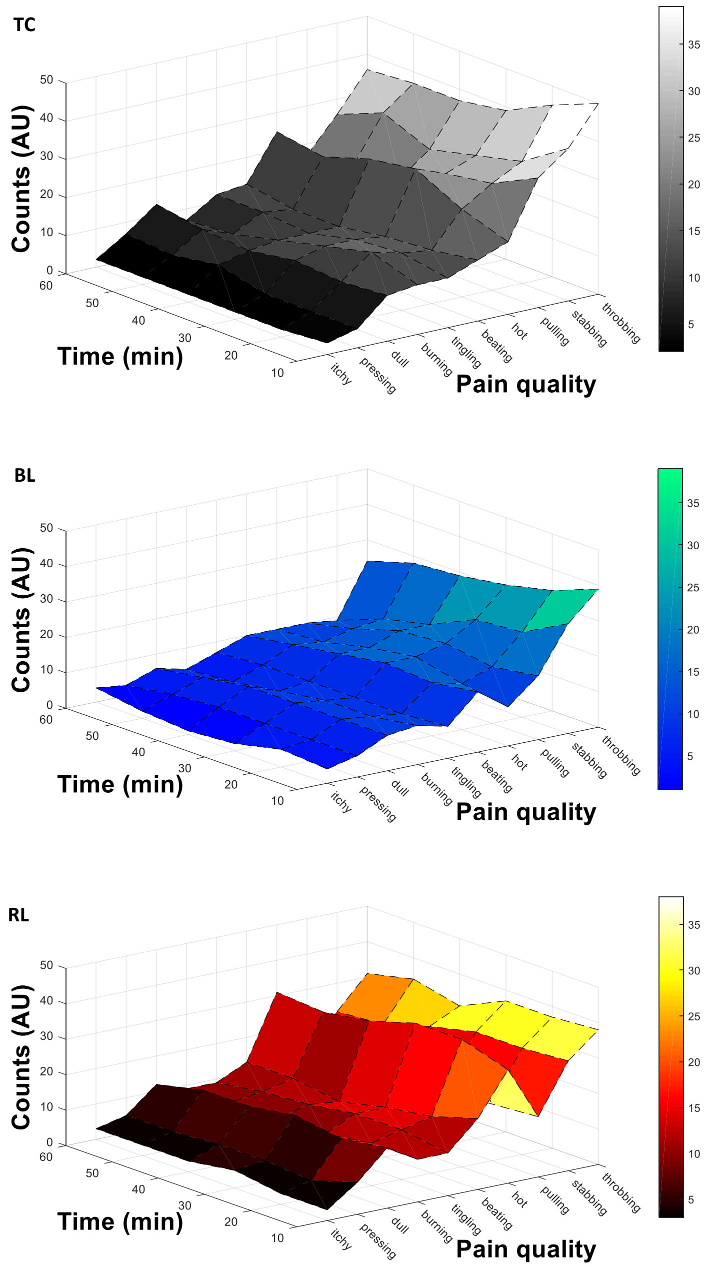

Supplementary Figure 2. Impact of treatment on pain quality in test arm.

Pain quality plots under treatment with TC (grey plot), BL (blue plot), and RL (red plot). *t* = 10 min represents the time point 10 min after treatment, *t* = 60 min the last time point of electrical stimulation and treatment. Data are represented as the sum of individually scored pain intensities (0-3) per quality (*n* = 27).

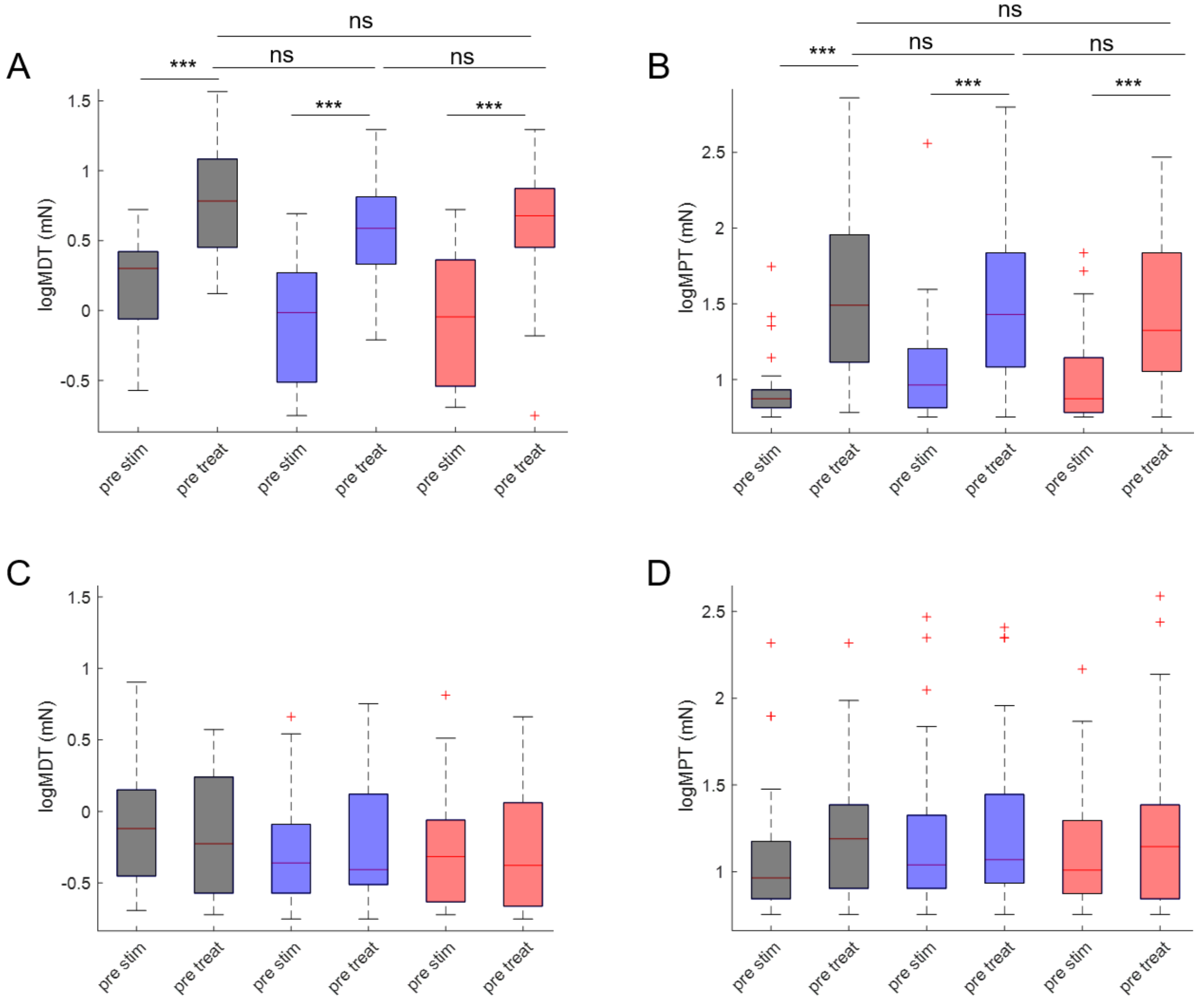

A
B
C
D
ns
***
ns
ns
***
***
logMDT (mN)
logMPT (mN)
pre stim
pre treat

Supplementary Figure 3. Impact of electrical stimulation on QST parameters in test and control arm.

(A) Logarithmic MDT of test arm in mN (*n* = 30) before (pre-stim) and after (pre-treat) stimulation onset, before treatment with TC (black boxes), BL (blue boxes), and RL (red boxes). All data are represented as median (red line) with interquartile range (box edges represent 25th and 75th percentiles) with extreme data points (boxplot whiskers) and outliers ('+' symbols). Nonparametric one-way-ANOVA for repeated measures (Friedman test) was calculated with Dunn's posttest. $^{ns}p > 0.05$, $^{***}p < 0.001$.

(B) Logarithmic MPT of test arm in mN (*n* = 30) before (pre-stim) and after (pre-treat) stimulation onset, before treatment with TC (black boxes), BL (blue boxes), and RL (red boxes). Nonparametric one-way-ANOVA for repeated measures (Friedman test) was calculated with Dunn's posttest. $^{ns}p > 0.05$, $^{***}p < 0.001$.

(C) Logarithmic MDT of unstimulated, untreated control arm in mN (*n* = 30) before (pre-stim) and after (pre-treat) stimulation onset at test arm, before treatment of test arm with TC (black boxes), BL (blue boxes), and RL (red boxes). Nonparametric one-way-ANOVA for repeated measures (Friedman test) was calculated with Dunn's posttest. $^{ns}p > 0.05$.

(D) Logarithmic MPT of unstimulated, untreated control arm in mN (*n* = 30) before (pre-stim) and after (pre-treat) stimulation onset at test arm, before treatment of test arm with TC (black boxes), BL (blue boxes), and RL (red boxes). Nonparametric one-way-ANOVA for repeated measures (Friedman test) was calculated with Dunn's posttest. $^{ns}p > 0.05$.

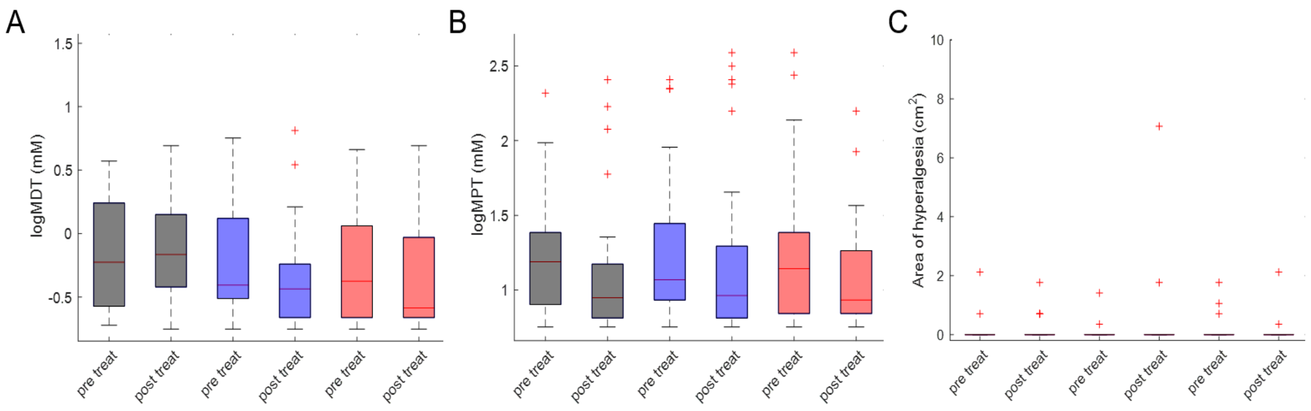

Supplementary Figure 4. Impact of treatment on QST parameters in control arm.

(**A**) Logarithmic MDT of unstimulated, untreated control arm in mN (*n* = 30) before (pre-treat) and after (post-treat) treatment of test arm with TC (black boxes), BL (blue boxes), and RL (red boxes). All data are represented as median (red line) with interquartile range (box edges represent 25th and 75th percentiles) with extreme data points (boxplot whiskers) and outliers ('**+**' symbols). Nonparametric one-way-ANOVA for repeated measures (Friedman test) was calculated with Dunn's posttest.

(B) Logarithmic MPT of unstimulated, untreated control arm in mN (*n* = 30) before (pre-treat) and after (post-treat) treatment of test arm with TC (black boxes), BL (blue boxes), and RL (red boxes). Nonparametric one-way-ANOVA for repeated measures (Friedman test) was calculated with Dunn's posttest.

(C) Area of hyperalgesia in cm^2 (*n* = 30) of unstimulated, untreated control arm before (pre-treat) and after (post-treat) treatment of test arm with TC, BL, and RL. Nonparametric one-way-ANOVA for repeated measures (Friedman test) was calculated with Dunn's posttest.

**Parameter and treatment**	**TC 1st vs. BL 1st Difference**	**TC 1st vs. RL 1st Difference**	**BL 1st vs. RL 1st Difference**
Temperature (C°)<br>ART-aligned TC treatment<br>Time point 1-11	0,5370(ns)<br>0,5270(ns)<br>-0,003029(ns)<br>-0,1530(ns)<br>-0,5030(ns)<br>0,7270(ns)<br>0,7870(ns)<br>0,4370(ns)<br>0,1870(ns)<br>0,1970(ns)<br>0,03698(ns)	0,3712(ns)<br>0,1612(ns)<br>0,2712(ns)<br>0,1312(ns)<br>-0,5388(ns)<br>0,06121(ns)<br>-0,1388(ns)<br>-0,02879(ns)<br>0,1012(ns)<br>0,001213(ns)<br>0,3312(ns)	-0,1658(ns)<br>-0,3658(ns)<br>0,2742(ns)<br>0,2842(ns)<br>-0,03576(ns)<br>-0,6658(ns)<br>-0,9258(ns)<br>-0,4658(ns)<br>-0,08577(ns)<br>-0,1958(ns)<br>0,2942(ns)
Temperature (C°)<br>ART-aligned BL treatment<br>Time point 1-11	-0,1530(ns)<br>0,1070(ns)<br>0,1670(ns)<br>-0,2130(ns)<br>-0,2930(ns)<br>-0,2230(ns)<br>-0,1830(ns)<br>-0,5530(ns)<br>-0,5230(ns)<br>-0,5530(ns)<br>0,2570(ns)	-0,2188(ns)<br>-0,008789(ns)<br>-0,09879(ns)<br>-0,2688(ns)<br>0,04121(ns)<br>-0,1188(ns)<br>-0,07878(ns)<br>-0,3688(ns)<br>-0,4588(ns)<br>-0,8088(ns)<br>0,2612(ns)	-0,06576(ns)<br>-0,1158(ns)<br>-0,2658(ns)<br>-0,05576(ns)<br>0,3342(ns)<br>0,1042(ns)<br>0,1042(ns)<br>0,1842(ns)<br>0,06424(ns)<br>-0,2558(ns)<br>0,004238(ns)
Temperature (C°)<br>ART-aligned RL treatment<br>Time point 1-11	0,2670(ns)<br>-0,003036(ns)<br>-0,5230(ns)<br>-0,4830(ns)<br>0,1570(ns)<br>0,2070(ns)<br>0,4270(ns)<br>0,1870(ns)<br>-0,2630(ns)<br>-0,06303(ns)<br>-0,5230(ns)	-0,3588(ns)<br>-0,2488(ns)<br>-0,4288(ns)<br>-0,3888(ns)<br>0,5812(ns)<br>0,8712(ns)<br>0,9312(ns)<br>0,09121(ns)<br>-0,3488(ns)<br>0,3312(ns)<br>0,3712(ns)	-0,6258(ns)<br>-0,2458(ns)<br>0,09425(ns)<br>0,09424(ns)<br>0,4242(ns)<br>0,6642(ns)<br>0,5042(ns)<br>-0,09576(ns)<br>-0,08576(ns)<br>0,3942(ns)<br>0,8942(ns)

Supplementary Table 1. Statistical comparison of test arm temperature dependent on initial treatment.

Statistical analysis of test arm temperature (n = 10, for each) for respective treatment (TC, BL, RL) dependent on initial treatment. Data were aligned-rank transformed and two-way-ANOVA with Bonferroni posttest was calculated. $^{ns}p > 0.05$.

Parameter and treatment	TC 1st vs. BL 1st Difference	TC 1st vs. RL 1st Difference	BL 1st vs. RL 1st Difference
Pain score (AU)<br>ART-aligned TC treatment<br>Time point 1-11	-2,139(ns)<br>-1,939(ns)<br>-2,139(ns)<br>-2,139(ns)<br>3,161(ns)<br>-4,239(ns)<br>0,6606(ns)<br>-2,839(ns)<br>-2,839(ns)<br>0,5606(ns)<br>-1,139(ns)	-2,230(ns)<br>-2,030(ns)<br>-2,230(ns)<br>-0,2303(ns)<br>1,070(ns)<br>-2,230(ns)<br>0,9697(ns)<br>1,570(ns)<br>-2,330(ns)<br>0,9697(ns)<br>-3,730(ns)	-0,09091(ns)<br>-0,09091(ns)<br>-0,09091(ns)<br>1,909(ns)<br>-2,091(ns)<br>2,009(ns)<br>0,3091(ns)<br>4,409(ns)<br>0,5091(ns)<br>0,4091(ns)<br>-2,591(ns)
Pain score (AU)<br>ART-aligned BL treatment<br>Time point 1-11	-1,639(ns)<br>-2,139(ns)<br>-2,139(ns)<br>-3,239(ns)<br>4,761(ns)<br>7,261(ns)<br>3,261(ns)<br>3,361(ns)<br>0,1606(ns)<br>-2,039(ns)<br>-2,139(ns)	-2,230(ns)<br>-2,730(ns)<br>-2,230(ns)<br>-3,330(ns)<br>5,570(ns)<br>6,170(ns)<br>3,370(ns)<br>3,570(ns)<br>1,970(ns)<br>1,770(ns)<br>-2,230(ns)	-0,5909(ns)<br>-0,5909(ns)<br>-0,09091(ns)<br>-0,09091(ns)<br>0,8091(ns)<br>-1,091(ns)<br>0,1091(ns)<br>0,2091(ns)<br>1,809(ns)<br>3,809(ns)<br>-0,09091(ns)
Pain score (AU)<br>ART-aligned RL treatment<br>Time point 1-11	-2,139(ns)<br>-2,139(ns)<br>-2,139(ns)<br>-0,1394(ns)<br>-0,1394(ns)<br>-0,7394(ns)<br>3,661(ns)<br>4,161(ns)<br>2,061(ns)<br>3,161(ns)<br>3,961(ns)	-2,230(ns)<br>-2,230(ns)<br>-2,230(ns)<br>-2,230(ns)<br>-2,330(ns)<br>-1,330(ns)<br>3,670(ns)<br>3,670(ns)<br>2,770(ns)<br>5,470(ns)<br>-2,230(ns)	-0,09091(ns)<br>-0,09091(ns)<br>-0,09091(ns)<br>-2,091(ns)<br>-2,191(ns)<br>-0,5909(ns)<br>0,009090(ns)<br>-0,4909(ns)<br>0,7091(ns)<br>2,309(ns)<br>-6,191(ns)

Supplementary Table 2. Statistical comparison of test arm pain score dependent on initial treatment.

Statistical analysis of test arm pain score (*n* = 10, for each) for respective treatment (TC, BL, RL) dependent on initial treatment. Data were aligned-rank transformed and two-way-ANOVA with Bonferroni posttest was calculated. $^{ns}p > 0.05$.

Parameter and treatment	TC 1st vs. BL 1st Difference rank sum	TC 1st vs. RL 1st Difference rank sum	BL 1st vs. RL 1st Difference rank sum
LogMDT(mM) TC treat	7.00 (ns)	-3.00 (ns)	-10.00 (ns)
LogMDT(mM) BL treat	14.50 (ns)	4.50 (ns)	-10.00 (ns)
LogMDT(mM) RL treat	7.00 (ns)	-5.00 (ns)	-12.00 (ns)
LogMPT(mM) TC treat	-1.00 (ns)	15.50 (ns)	16.50 (ns)
LogMPT(mM) BL treat	-0.50 (ns)	3.00 (ns)	3.50 (ns)
LogMPT(mM) RL treat	-14.50 (ns)	2.50 (ns)	17.00 (ns)
Area of flare TC treat	11.00 (ns)	11.00 (ns)	0.00 (ns)
Area of flare BL treat	-11.50 (ns)	-11.00 (ns)	0.50 (ns)
Area of flare RL treat	9.50 (ns)	-19.50 (ns)	-29.00 (**)
Area of hyperalgesia TC treat	-19.50 (ns)	-15.50 (ns)	4.00 (ns)
Area of hyperalgesia BL treat	-0.05 (ns)	1.00 (ns)	1.50 (ns)
Area of hyperalgesia RL treat	-14.50 (ns)	7.50 (ns)	22.00 (ns)
Area of allodynia TC treat	-7.00 (ns)	3.50 (ns)	10.50 (ns)
Area of allodynia BL treat	2.00 (ns)	2.00 (ns)	0.00 (ns)
Area of allodynia RL treat	-8.00 (ns)	7.50 (ns)	15.50 (ns)

Supplementary Table 3. Statistical comparison of test arm MDT, MPT, area of flare, area of hyperalgesia, and area of allodynia dependent on initial treatment.

Statistical analysis of QST parameters (n = 10, for each) for respective treatment (TC, BL, RL) dependent on initial treatment. Nonparametric one-way-ANOVA for repeated measures (Friedman test) was calculated with Dunn's posttest. $^{ns}p > 0.05$, $^{**}p < 0.01$.
